# Circulating Tumor Cells and Breast Cancer Metastasis: From Enumeration to Somatic Mutational Profile

**DOI:** 10.3390/jcm11206067

**Published:** 2022-10-14

**Authors:** Chengjun Zhu, Jing Xu, Jinyu Sun, Shiyun Cui, Yue Sun, Tao Yu, Cenzhu Wang, Tianyao Wang, Yufeng Wu, Feng Ju, Jiafeng Yao, Kai Liu, Wenwen Zhang, Xiaoxiang Guan

**Affiliations:** 1 Department of Oncology, The First Affiliated Hospital of Nanjing Medical University, No. 300 Guangzhou Road, Nanjing 210029, China; 2 Stomatological College, Nanjing Medical University, Nanjing 210029, China; 3 College of Mechanical and Electrical Engineering, Nanjing University of Aeronautics and Astronautics, Nanjing 210016, China; 4 Department of Oncology, Nanjing First Hospital, Nanjing Medical University, 68 Changle Road, Nanjing 210006, China

**Keywords:** circulating tumor cells, breast cancer, metastasis, liver metastasis, biomarker

## Abstract

Aims: This study investigates the association between circulating tumor cells (CTCs) and breast cancer metastasis. Methods: A retrospective study was conducted using patients with histologically confirmed breast cancer recruited from the First Affiliated Hospital of Nanjing Medical University during the period of August 2017–October 2020. We used adjusted logistic regression, the random forest algorithm, and sensitivity analysis to study the association between CTC enumeration and tumor metastasis. Further, we performed next-generation sequencing (NGS) on the CTCs obtained from two patients with breast cancer brain metastasis. Results: A total of 41 out of 116 enrolled patients were identified with tumor metastasis. CTC enumeration was significantly higher in patients with liver metastasis than in those without liver metastasis. Patients with CTCs ≥ 5 exhibited a higher risk of tumor metastasis than those with CTCs < 5 in the adjusted model (odds ratios (OR) = 6.25, 95% confidence interval (CI) = 2.63–15.58). The random forest model identified CTC enumeration as a significant metastasis-related variable with the highest mean decrease accuracy and mean decrease Gini score. No significant association was found between CTCs and visceral metastasis with an OR of 1.29 (95% CI = 0.98–2.05, *p* = 0.232). Upon further investigating organ-specific metastasis, we found that patients with high CTC levels were more likely to develop liver metastasis (OR = 4.87, 95% CI = 1.34–20.17, *p* = 0.021). The NGS study of CTCs identified a total of 120 indel mutations (e.g., *CNGB1*, *NTSR1*, *ZG16*). The enriched biological processes were mechanoreceptor differentiation and macrophage activation involved in the immune response. The enriched KEGG pathways included focal adhesion, the PI3K-Akt signaling pathway, and microRNAs involved in cancer. Conclusions: Our study revealed that CTCs ≥ 5 are a risk factor for tumor metastasis in breast cancer patients. In addition, we reported that CTCs ≥ 5 might be associated with a higher risk of liver metastasis in patients with metastatic breast cancer. We have provided the mutational profiles of CTCs based on next-generation sequencing.

## 1. Introduction

Breast cancer is the most common malignancy worldwide, accounting for about 11.7% of all cancers [1] and 15% of cancer-associated deaths [2]. Despite the technology and clinical advances, breast cancer metastasis and related complications remain a major cause of death. About 12% of breast cancer patients develop distant metastasis, reducing the 5-year survival rate drastically from over 90% to 25% [2,3]. Hence, it is important to identify patients at a high risk of tumor metastasis to provide just-in-time intervention.

Circulating tumor cells (CTCs) are cancer cells that originate from primary/metastatic tumors and circulate in the bloodstream [4]. After entering the blood circulation, the viable CTCs could go into the systemic circulation, extravasate through the vascular wall, and eventually lead to metastatic lesions in distant organs [5]. The presence of CTCs is closely associated with tumor micro-metastasis and is recognized as a precursor of distant metastasis [6]. Accumulating evidence from different studies has demonstrated that CTC enumeration could predict therapeutic response, disease progression, and overall survival in metastatic breast cancer [7,8,9,10]. Owing to the development of isolation techniques [11], CTCs can now be detected with high accuracy and reproducibility, facilitating CTC enumeration as a promising, minimally invasive method to monitor the progression of breast cancer [12]. CTC enumeration has been recently proposed for prognostic stratification in patients with metastatic breast cancer [13]. However, only a few studies have investigated the association between CTC enumeration and metastasis sites [12,14].

This study aims to investigate the association of CTC enumeration with the organ-specific metastasis of breast cancer. Further, we explore a somatic mutational landscape of CTCs based on next-generation sequencing.

## 2. Methods

### 2.1. Literature Review

A literature search was performed in PubMed database from 2010 to mid-2022 based on the following search strategy: ((Circulating tumor cell [Title/Abstract]) AND (cancer [Title/Abstract] OR tumor [Title/Abstract])) AND (metastasis [Title/Abstract]). The literature review was done on 5 April 2022.

### 2.2. Study Design

Female patients with histologically confirmed breast cancer were retrospectively recruited from the First Affiliated Hospital of Nanjing Medical University during the period August 2017–October 2020. We performed radiographic examinations (chest and abdomen computed tomography and brain magnetic resonance imaging) on all patients to assess distant metastasis. On the indication of clinical symptoms or radiographic evidence for bone metastasis, Emission Computed Tomography was conducted. Patients with liver, brain, or lung cancer were termed with visceral metastasis.

A routine pathologic evaluation was conducted to determine the status of primary tumor biomarkers, including estrogen receptor (ER), progesterone receptor (PR), human epidermal growth factor receptor 2 (HER2), and Ki67. Patients with HER2 of ++/+++ phenotype were identified as HER2-positive in this study. In addition, molecular subtypes were classified as HER2-positive, luminal (A and B with HER2-negative), and triple-negative breast cancer (TNBC). Due to the retrospective and fully anonymized nature of the data, informed consent was waived. This study was approved by the ethics committee of the First Affiliated Hospital of Nanjing Medical University.

### 2.3. Specimen Collection and CTC Isolation

Peripheral blood samples were collected before and after the systemic therapy of patients with and without tumor metastasis, respectively. CTCs were isolated by the CTCBIOPSY^®^ system following the manufacturer’s protocol (Wuhan YZY Medical Science and Technology Co., Ltd., Wuhan, China) [15,16]. The initial 1 mL of blood was discarded to avoid the contamination of epithelial cells. A 5 mL volume of blood was collected and diluted to 8 mL with 0.2% paraformaldehyde to maintain the cell morphology. Then, the mixture was transferred to a centrifuge tube, aerated, and absorbed by the Pasteur tube. After incubation at room temperature for 10 min, the blood sample was filtered through an 8 µm filter membrane by applying a pressure of 5 kilopascals. The residual cells remaining on the filter membrane were stained by Wright–Giemsa stain and dried at room temperature. The filter membrane was also dried at 50–60 °C for 30 min by adhering it to a slide. Finally, the slide was sealed, and CTCs were counted independently by two senior cytopathologists following a published method [17]. Any disagreement regarding the CTC enumeration was resolved by consulting a third cytopathologist.

### 2.4. Next-Generation Sequencing of CTC

We performed whole genomic amplification and next-generation sequencing (NGS) on CTCs collected from the two patients. Patient 1 was a 75-year-old female who was pathologically diagnosed with breast cancer brain metastasis, and a total of 7 CTCs were identified from her sample. Patient 2 was a 56-year-old female who was diagnosed with breast cancer bone metastasis, and 10 CTCs were identified from her sample. The identified CTCs were located and then sheared by laser capture microdissection. CTCs contained in the sampling needle were collected in a PCR tube, and whole-genome amplification was performed by multiple annealing and looping-based amplification cycle. Further, we used the NanoDrop^TM^ 2000 (Thermo Fisher, Wilmington, NC, USA) for quantitative analysis and performed NGS by the MGISEQ-T7(MGI, Shenzhen, China) according to the manufacturer’s protocols. Genetic information analysis was carried out based on the COSMIC database to identify indel mutations associated with breast cancer [18]. In the end, we performed Gene Ontology (GO) [19] and Kyoto Encyclopedia of Genes and Genomes (KEGG) [20] pathway enrichment analysis on the co-indel mutations using the cluster profiler package.

### 2.5. Statistical Analysis

We used multivariate multiple imputation strategies for the imputation of missing covariates to improve the statistical power and reduce the selection bias [21]. We evaluated normality by the Kolmogorov–Smirnov test and presented continuous variables as mean ± standard deviation (normal distribution) and median with interquartile range (skewed distribution). Categorical variables were represented in percentages. Patient characteristics were compared between the metastasis or non-metastasis groups using a one-way ANOVA test (normal distribution), Kruskal–Wallis test (skewed distribution), or chi-squared test (categorical variables).

We applied a logistic regression model to evaluate the association between CTC enumeration and tumor metastasis and calculate the odds ratios (ORs) with 95% confidence intervals (CIs). HER2 status was adjusted for covariates in the adjusted model. Additionally, we also explored if patients with CTCs ≥ 5 were at a higher risk of metastasis than those with CTCs < 5. We further investigated the association between CTC enumeration and metastatic sites in patients with metastatic breast cancer.

We used the random forest machine-learning algorithm to assess the significance of metastasis-related characteristics, including CTC enumeration, age, biomarker status, molecular subtypes, primary tumor side (left or right side), and the number of axillary lymph node metastases. This algorithm uses mean decrease accuracy and mean decrease Gini score in the feature assessment of variables. Mean decrease accuracy quantifies the decrease in accuracy of the model when excluding a specific variable, whereas the mean decrease Gini score measures the contribution of each variable to the homogeneity of the model. A higher value of mean decrease accuracy and mean decrease Gini index indicates a significant association between the variable and tumor metastasis. Thereby, we assessed the diagnostic performance of CTC enumeration for tumor metastasis or liver metastasis by calculating the area under the curve (AUC), sensitivity, specificity, positive predictive value, and negative predictive value.

Furthermore, we performed a sensitivity analysis to evaluate the association with different subgroups, including ER (positive or negative), PR (positive or negative), HER2 (positive or negative), Ki67 (<median or ≥median), and molecular subtypes (luminal, HER2, or triple-negative breast cancer). All statistical analyses were performed in R software (version 4.1.1, R Core Team). *p* < 0.05 was considered statistically significant.

## 3. Results

### 3.1. Literature Review

We reviewed a total of 378 research papers in the PubMed database from 2010 to mid-2022 and observed an increasing trend in the number of publications, with an annual growth rate of 12.25%. We summarized the recent studies on the association between CTCs and breast cancer metastasis in Table 1 [14,22,23,24,25,26,27,28].

### 3.2. Patient’s Characteristics

A total of 116 patients were enrolled in this study, and tumor metastasis was found in 41 of them. Compared to patients with non-metastatic tumors, patients with tumor metastasis showed a higher CTC enumeration (5 vs. 2, *p* < 0.001), larger tumor size (2.5 vs. 2.0, *p* = 0.007), more axillary lymph node metastasis (2 vs. 0, *p* = 0.01), and higher HER2-positive status (56.1% vs. 34.7%, *p* = 0.042). No significant differences were observed in age, ER, PR and Ki67 status, and molecular subtype (all *p* > 0.05). Detailed patient characteristics are tabulated in Table 2.

**Figure 1:**
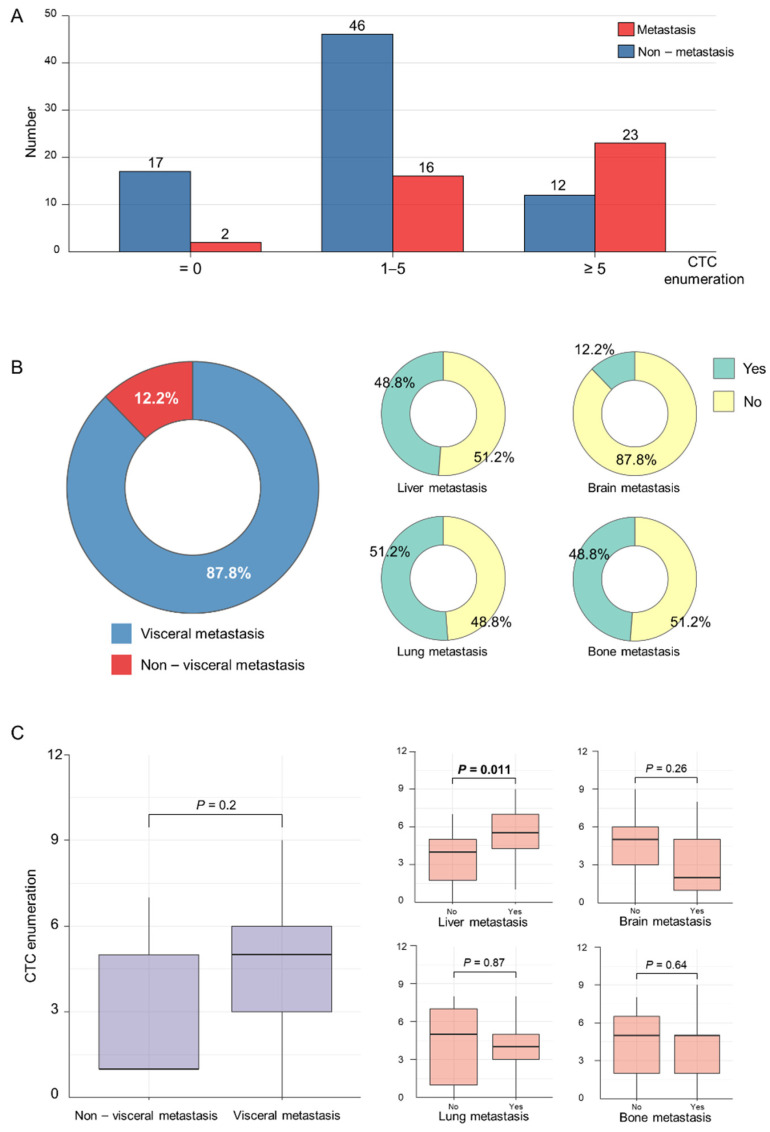
Circulating tumor cells enumeration and tumor metastasis site. (**A**) The number of patients across different CTC enumeration categories (CTCs = 0, 1–5, and ≥5). (**B**) The constituent ratio of organ-specific metastasis in patients with metastatic breast cancer. Constituent ratios are shown in visceral, liver, brain, lung, and bone metastasis, respectively. (**C**) CTC enumeration in different metastasis sites. CTC enumeration in different groups was compared by the Wilcox test. The bold format means statistics significant. Abbreviation: CTC—circulating tumor cell.

We illustrated the number of patients in different CTC enumeration categories (CTCs = 0, 1–5, and ≥ 5). As shown in Figure 1A, 97 patients had CTCs ≥ 1, and 35 patients had CTCs ≥ 5. More non-metastasis cancer was observed in patients with CTCs < 5 while more metastatic breast cancer was observed in the subgroup of CTC enumeration ≥ 5. We further analyzed the constituent ratio of organ-specific metastasis in patients with metastatic cancer (Figure 1B). Visceral metastasis was observed in most patients with a constituent ratio of 87.8%, whereas liver, lung, and bone metastasis was observed in almost 50% of the individuals. Besides, we illustrated and compared the CTC enumeration in different metastatic sites by boxplots (Figure 1C). Importantly, CTC enumeration was found to be significantly higher in patients with liver metastasis compared to those without liver metastasis (Wilcox test *p* = 0.011).

### 3.3. The Association between CTC Enumeration and Tumor Metastasis

**Figure 2:**
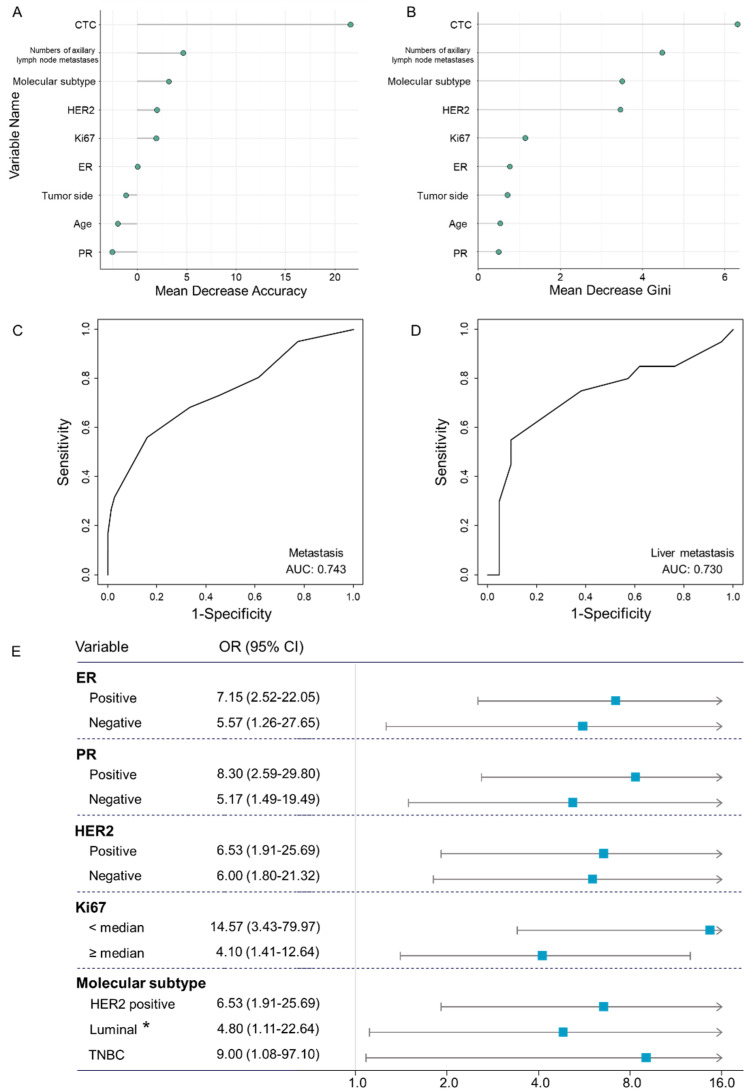
The evaluation of variable importance and diagnostic performance. A random forest algorithm was used to calculate (**A**) mean decrease accuracy and (**B**) mean decrease Gini score. A higher mean decrease accuracy and mean decrease Gini index indicates a more important association between the variable and tumor metastasis. CTC enumeration showed the highest variable importance with a mean decrease accuracy of 21.5 and a mean decrease Gini score of 6.4. Receiver operating characteristic curve of CTC enumeration for (**C**) tumor metastasis in patients with breast cancer and (**D**) liver metastasis in patients with metastatic breast cancer. (**E**) Forest plot of sensitivity analysis for the positive association between CTC enumeration and tumor metastasis across ER (positive or negative), PR (positive or negative), HER2 (positive or negative), Ki67 (<median or ≥median), and molecular subtypes (HER2 positive, luminal, or triple-negative breast cancer). The odds ratios with 95% confidence intervals were calculated and the group of CTCs < 5 was set as the reference. * means Luminal A and B with HER2-negative. Abbreviation: AUC—area under the curve; CI—confidence interval; CTC—circulating tumor cell; ER—estrogen receptor; HER2—human epidermal growth factor receptor 2; OR—odds ratio; PR—progesterone receptor; TNBC—triple-negative breast cancer.

When CTC enumeration was analyzed as a continuous variable, it was found to be significantly associated with tumor metastasis in both crude and adjusted models with ORs of 1.50 (95% CI, 1.23–1.86) and 1.50 (95% CI, 1.23–1.88), respectively (Table 3). Patients with CTCs ≥ 5 showed a higher risk of tumor metastasis relative to those with CTCs < 5 in the adjusted model (OR = 6.25; 95% CI, 2.63–15.58; *p* < 0.001). In addition, we used the random forest algorithm to further evaluate the association. CTC enumeration exhibited the highest variable importance with a mean decrease accuracy of 21.5 and a mean decrease Gini score of 6.4 (Figure 2A,B).

Moreover, we explored the association between CTC enumeration and organ-specific metastasis of viscera, liver, brain, lung, and bones (Table 4). No significant association was observed in visceral metastasis with an OR of 1.29 (95% CI = 0.98–2.05; *p* = 0.232). Interestingly, we found that patients with CTCs ≥ 5 were more likely to develop liver metastasis (OR = 4.87; 95% CI = 1.34–20.17; *p* = 0.021).

Furthermore, we performed a diagnostic analysis to test whether CTC enumeration could be used as a biomarker of tumor metastasis in breast cancer patients. The receiver operating characteristic curve indicated a reliable performance with an AUC of 0.743, a sensitivity of 0.561, a specificity of 0.840, a positive predictive value of 0.657, and a negative value of 0.778 (Figure 2C). Furthermore, we also evaluated the diagnostic performance of CTC enumeration for liver metastasis in patients with metastatic breast cancer. The results showed an AUC of 0.730, a sensitivity of 0.550, a specificity of 0.905, a positive predictive value of 0.856, and a negative value of 0.679 (Figure 2D).

As illustrated in Figure 2E, the positive association between CTC enumeration and tumor metastasis remained stable across ER (positive or negative), PR (positive or negative), HER2 (positive or negative), Ki67 (<median or ≥median), and molecular subtypes (HER2 positive, luminal, or triple-negative breast cancer).

### 3.4. Somatic Mutational Landscape in CTCs

**Figure 3:**
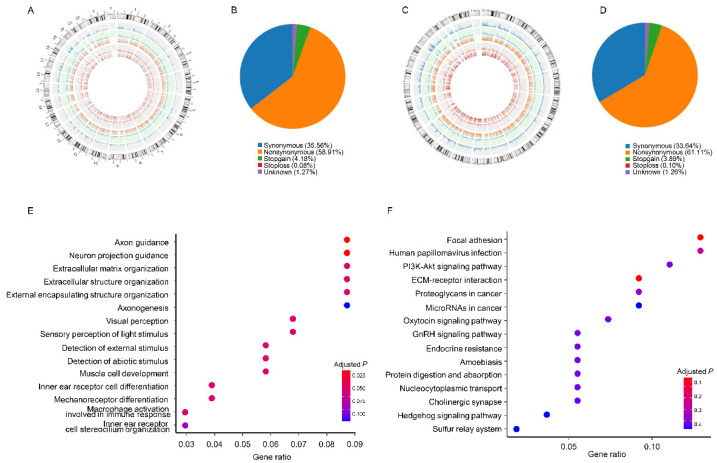
Mutation of circulating tumor cells detected by next-generation sequencing. For patient 1: (**A**) The overview of the somatic mutational profiles of CTCs. Chromosomes are presented in the outermost circle clockwise. The blue histograms indicate the log-transformed sequencing depth, setting 1 Mbp as a unit. The blue points show the number of SNPs in each chromosome. The following orange histograms suggest the relative proportion of homologous SNPs; the grey area in this ring refers to heterozygous SNPs. The red points stand for the number of indel mutations in each chromosome. The innermost circle shows the proportion of homologous or heterozygous indel mutations; red histograms refer to homologous mutations and the grey area refers to heterozygous mutations. (**B**) The distribution of CTC mutations. For patient 2. (**C**) The overview of the somatic mutational profiles of CTCs. (**D**) The distribution of CTC mutations. (**E**) Gene ontology biological process, and (**F**) KEGG pathway enrichment analysis in circulating tumor cells. Abbreviation: SNP—single-nucleotide polymorph.

For patient 1, Figure 3A provides an overview of the somatic mutation profiles of CTCs, which shows sequencing depth, SNPs number, indel mutation number, and homologous/heterozygous mutation proportion. As shown in Figure 3B, most of the mutations were nonsynonymous (58.91%) followed by synonymous (35.56%). The somatic mutation profiles of patient 2 are shown in Figure 3C. Consistent with patient 1, most mutations detected in patient 2 were also nonsynonymous (58.91%) followed by synonymous, which account for about 35.56% (Figure 3D). The details of indel mutations in patient 1 and patient 2 are presented in Table S1 and Table S2, respectively. After overlapping, the NGS analysis of CTCs identified a total of 120 indel mutations, including CNGB1, NTSR1, ZG16, and many more (Table S3). Figure 3E shows the enriched biological processes such as axon guidance, extracellular matrix organization, mechanoreceptor differentiation, and macrophage activation involved in the immune response. The enriched KEGG pathways included focal adhesion, PI3K-Akt signaling pathway, and proteoglycans and microRNAs in cancer (Figure 3F).

## 4. Discussion

Although CTCs were first reported in 1869, the challenge of their low concentration and isolation technique blocked their clinical application. Over the past two decades, the advances in detection methods have facilitated the sophisticated isolation of CTCs, allowing a more in-depth investigation of them as a biomarker for tumor progress. Since CTCs are a mixture of cells from multiple tumor regions, their biopsy, unlike traditional methods, provides a minimally invasive method for a more comprehensive view of intra-tumor heterogeneity [29]. Recently, a pooled analysis of 1944 patients with metastatic breast cancer demonstrated the effect of CTC enumeration as an independent predictor for overall and progression-free survival [30]. Clinical evidence shows that CTC enumeration might be an earlier and more accurate method for predicting the overall survival of metastatic breast cancer patients than radiology methods [31]. Therefore, CTC enumeration has now become one of the cornerstones of real-time liquid biopsy and has been used as a minimally invasive, convenient, and patient-friendly biomarker to monitor tumor metastasis, its recurrence, and therapeutic responses [32,33,34]. The serial analysis of CTCs might provide valuable information for metastasis detection and treatment decisions [35,36,37,38].

Our study provided evidence to support CTC enumeration as a potential biomarker of tumor metastasis in breast cancer patients. CTCs ≥ 5 were identified in about 50% of patients with metastatic breast cancer. Our study confirmed the presence of CTCs ≥ 5 as an effective biomarker of tumor metastasis in patients with breast cancer, similar to previous trials [27,39,40]. The sensitivity analysis showed that the association between CTC enumeration and metastasis remained stable in different molecular subtypes. Similar to a recent real-world study by Costa et al. [12], we also observed no significant association between CTC enumeration and visceral or non-visceral metastasis.

The livIr is one of the most common organs for breast cancer metastasis. Liver metastasis has been observed in about 40–50% of patients diagnosed with metastatic breast cancer [41]. Interestingly, our study reported that patients with high CTC levels are more likely to develop liver metastasis upon the investigation of organ-specific metastasis. CTC enumeration was found to be significantly higher in patients with liver metastasis than in those without. The logistic regression showed an increased risk of liver metastasis in patients with CTCs ≥ 5. A previous study using Pep@MNPs (peptide-based nanomagnetic CTC isolation) system also suggested that patients with > 2 CTCs/2 mL of blood are more likely to develop liver metastasis (*p* = 0.01) [28]. In contrast, the study by Giorgi and colleagues [14] reported that CTC enumeration is significantly higher in patients with bone metastasis but not liver metastasis. This study included 195 patients with relapsed and progressive metastatic breast cancer. Bone metastasis was observed in about 70% of the patients, while liver metastasis was observed in only 31%. The difference in patient inclusion criteria might contribute to the inconsistent results. However, CTC enumeration was still higher in patients with liver metastasis than in those with lung, pleural, or soft-tissue metastasis [14]. Our results revealed that the circulation and invasion of CTCs might show distinct characteristics in the liver, which indicates the potential significance of CTCs in detecting breast cancer liver metastasis. However, the mechanisms underlying high CTC enumeration-related liver metastasis remain to be explored.

Besides enumeration, the biological phenotype of CTC provides an added value to its malignant behavior. In our previous study, we used a “seed and soil” model to explore the association between the heterogeneity and organotropism of CTCs in breast cancer [42]. Compared with other cancer cells in the tumor, CTCs showed a higher level of inter-/intra-patient heterogeneity [43]. CTCs can be classified into subgroups with distinct biological features based on biomarker status, epithelial/mesenchymal phenotype, and aggregation status [42]. It would be interesting to investigate the association between the heterogeneity of CTCs and organ-specific metastasis. The present study evaluated the molecular characterization of CTCs by NGS and provided an overview of the somatic mutational landscape of CTCs. A total of 379 point or indel mutations were detected in CTCs, including *ATAD3B*, *GPR153*, *MTOR*, and more. We observed NTSR1 mutation in both patients, which indicated that NTSR1 might be a potential candidate for tumor progression. It was reported that 91% of invasive ductal breast carcinoma specimens expressed NTSR1, and NTSR1 was involved in cellular migration, invasion, and the induction of matrix metalloproteases-9 [44]. An NTSR1 antagonist SR48692 could hinder tumor growth in triple-negative cancer cells (MDA-MB-231) xenografted in nude mice [44]. Our results indicated that NTSR1 mutation might be a potential new target for the treatment of triple-negative breast cancer. Therefore, it is interesting to explore the effects of NTSR1 mutation in triple-negative breast cancer cell lines on proliferation, migration, invasion, and the response to antagonist SR48692. Moreover, the gene ontology biological processes enriched macrophage activation in the immune response. Previous studies have reported that large numbers of tumor-associated macrophages were observed in many malignancies, which can promote tumor angiogenesis, induce CTCs releasing, and inhibit anti-tumor immunity [45]. Macrophage-based tumor immunotherapy methods have also been recently developed, such as chimeric antigen receptor macrophage cell therapy [46]. The enriched KEGG pathways included the PI3K-Akt signaling pathway. It would be interesting to co-culture PIK3CA inhibitors with CTCs isolated from the peripheral blood of the two patients in vitro to observe whether the CTC growth is inhibited and develop treatment strategies based on CTC gene expression. Our results suggest that combining CTC enumeration, molecular characterization, and other clinical variables (e.g., age, tumor stage, biomarker status, molecular subtypes) might provide an accurate tool for evaluating cancer metastasis risk [47,48].

Cancer treatment has now become increasingly individualized, and the application of CTC to cancer treatment and prognosis management could potentially move personalized medicine forward to the next step [49]. Many trials have already been completed or are now investigating the application of CTCs in treatment decisions against early breast cancer (such as the TREAT-CTC study [50]) and metastatic breast cancer (such as SWOG S0500 [51], CirCe01 [52], STIC CTC [53], CirCe T-DM1 [54], and the DETECT Study Program [55]). In a recent prospective study on 67 patients with metastatic breast cancer [56], the DNA end-binding protein p53-binding protein 1 (53BP1) levels in CTCs were significantly higher in patients with hormone receptor-positive metastases, especially following chemotherapeutic treatment by Eribulin. Kaplan–Meier analysis also revealed that nuclear 53BP1-positivity was associated with an increasing progression-free survival [56]. Moreover, Trapp et al. [57] reported that breast cancer patients with CTCs were likely to have bone-only first distant disease and first distant disease at multiple than those patients without CTCs. The accumulating evidence reveals the clinical potential and utility of CTCs.

Despite the novel insights provided by this study into CTCs and tumor metastasis, some limitations should be noticed. First, only 116 patients with breast cancer were included, with only 41 participants diagnosed with metastatic tumors. The small sample size limits the statistical power of this study. A further investigation of a larger population is necessary to validate our results. Second, most of the patients were older than 40 years. Thus, it is uncertain if the findings can be applied to young individuals. Third, the composition of patients included in this study was heterogeneous in biomarker status, molecular subtypes, and therapies. Although most of the current studies are based on carefully designed trials including homogeneous individuals, our study could provide additional clinical evidence for CTC enumeration in evaluating tumor metastasis. Fourth, the cross-sectional study design makes it difficult to assess the CTC enumeration value in predicting tumor metastasis. Although distant metastasis was evaluated by radiographic examination, we are unclear about the undetected metastatic lesions in patients with high CTCs. In the following research, the comparison of somatic mutations between CTCs and primary breast cancer and the association between molecular characterization and breast cancer metastasis should be investigated.

## 5. Conclusions

Our study highlighted that CTCs ≥ 5 are a risk factor for tumor metastasis in patients with breast cancer, and the association remained robust in different molecular subtypes. CTC enumeration was found to be high in patients with breast cancer liver metastasis relative to those without. The presence of CTCs ≥ 5 is a biomarker for the increased risk of liver metastasis in patients with metastatic breast cancer. We also provided the somatic mutational profiles of CTCs based on the NGS analysis. More studies are required to further validate the prognostic value of CTC enumeration for liver metastasis in patients with breast cancer.

## Figures and Tables

**Table 1:** Recent studies on the association between CTCs and breast cancer metastasis.

CTC Signature	Primary Results	Sample Sizes	Ref.
CTC enumeration	50% of the patients with metastatic breast cancer were identified as having 2 CTCs per 7.5 mL of blood.	38 mBC	[27]
CTC enumeration	Significantly higher CTC enumeration was detected in patients with bone metastasis than in those with no bone lesions.	195 mBC	[14]
CTC enumeration	High CTC enumeration (>2 per 2 mL of blood) at baseline was more likely to develop liver metastasis.	102 mBC	[28]
CTC enumeration	High CTC enumeration (>15 per 7.5 mL of blood) was correlated with disease severity and metastatic progression for breast cancer with liver metastasis.	43 mBC	[22]
CTC enumeration	High CTCs were significantly associated with lymph node metastasis in patients with breast cancer.	128 BC	[23]
Molecular characterization	Breast cancer brain metastasis had twice as many Ki67High CTCs (ratio of Ki67High: Ki67Low = 2:1).	10 BC	[24]
Molecular characterization	Increased FADS3 expression contributed to lung metastasis formation.	44 BC	[25]
CTC enumeration and molecular characterization	GM^+^(DAPI^+^CD45^–^PGK1/G6PD^+^) CTC enumeration was significantly correlated with breast cancer metastasis and progression.	64 BC	[26]

Abbreviation: mBC—metastatic breast cancer; BC—breast cancer.

**Table 2:** Patient’s characteristics.

	Overall (*n* = 116)	Metastasis (*n* = 41)	Non-Metastasis (*n* = 75)	*p*-Value
**Molecular subtype (%)**				0.072
HER2-positive	49 (42.2)	23 (56.1)	26 (34.7)	
Luminal *	41 (35.3)	12 (29.3)	29 (38.7)	
TNBC	26 (22.4)	6 (14.6)	20 (26.7)	
**Tumor size (cm)**	2.2 [1.7, 3.0]	2.5 [2.0, 3.9]	2.0 [1.5, 2.5]	**0.007**
**Number of axillary lymph node metastases**	1 [0, 3]	2 [0, 6]	0 [0, 2]	**0.010**
**CTCs**				
CTC enumeration (continuous)	3 [1, 5]	5 [2, 7]	2 [1, 4]	**<0.001**
CTC enumeration < 5, *n* (%)	81 (69.8)	18 (43.9)	63 (84.0)	**<0.001**
CTC enumeration ≥ 5, *n* (%)	35 (30.2)	23 (56.1)	12 (16.0)	**<0.001**

* Luminal A and B with HER2-negative. The bold format means statistics significant. Abbreviation: CTCs—circulating tumor cells; ER—estrogen receptor; PR—progesterone receptor; HER2—human epidermal growth factor receptor 2; TNBC—triple-negative breast cancer.

**Table 3:** Association of circulating tumor cell enumeration with tumor metastasis.

	Non-Adjusted Model		Adjusted Model	
	Odds Ratio	*p*-Value	Odds Ratio	*p*-Value
CTC enumeration (per unit)	1.50 (1.23–1.86)	**<0.001**	1.50 (1.23–1.88)	**<0.001**
Categories				
CTC enumeration < 5	*Reference*		*Reference*	
CTC enumeration ≥ 5	6.71 (2.86–16.55)	**<0.001**	6.25 (2.63–15.58)	**<0.001**

The bold format means statistics significant. Abbreviation: CTC—circulating tumor cell.

**Table 4:** Association between circulating tumor cell enumeration and metastasis sites.

	CTC Enumeration (per Unit)		CTC Enumeration ≥ 5 *	
Metastasis Site	Odds Ratio	*p*-Value	Odds Ratio	*p*-Value
Viscera	1.29 (0.98–2.05)	0.232	2.10 (0.31–17.47)	0.446
Liver	1.02 (0.96–1.10)	0.560	4.87 (1.34–20.17)	**0.021**
Brain	0.81 (0.52–1.04)	0.304	0.48 (0.06–3.21)	0.446
Lung	1.02 (0.96–1.11)	0.560	0.73 (0.21–2.53)	0.624
Bone	1.00 (0.94–1.07)	0.999	0.92 (0.26–3.17)	0.890

* The group of CTCs < 5 was set as the reference. The bold format means statistics significant.

## Data Availability

The data that support the findings of this study are available from the corresponding author upon reasonable request.

## Supplementary Materials

The following supporting information can be downloaded at: https://www.mdpi.com/article/10.3390/jcm11206067/s1, Table S1: The indel mutation profiles of CTCs for patient 1; Table S2: The indel mutation profiles of CTCs for patient 2; Table S3: The overlapped indel mutation profiles of CTCs.
